# Does the Silver Nanoparticles Production Route Affect the Proliferation of Antibiotic Resistance in Soil Ecosystem?

**DOI:** 10.3390/antibiotics14010015

**Published:** 2024-12-29

**Authors:** Jana Sedlakova-Kadukova, Miroslava Sincak, Veronika Demčakova

**Affiliations:** 1 Institute of Chemistry and Environmental Sciences, Faculty of Natural Sciences, Ss. Cyril and Methodius University in Trnava, Nam. J. Herdu 2, 917 01 Trnava, Slovakia; jana.sedlakova.fpv@ucm.sk; 2 ALGAJAS s.r.o., Prazska 16, 040 11 Kosice, Slovakia; 3 Faculty of Natural Science, Pavol Jozef Safarik University in Kosice, Srobarova 2, 041 54 Kosice, Slovakia; vdweronika@gmail.com

**Keywords:** silver nanoparticles, antibiotic tolerance, metal tolerance

## Abstract

Introduction: Silver nanoparticles (AgNPs) are widely utilized for their exceptional antimicrobial properties, but concerns persist regarding their environmental impacts, particularly in soil and water ecosystems. This study compared the effects of chemically and biologically synthesized AgNPs and ionic silver on bacterial communities commonly present in soil and the proliferation of antibiotic resistance in the soil ecosystem. Results and Discussion: Biologically synthesized AgNPs exhibited the strongest antimicrobial activity, significantly reducing bacterial populations within a day, and demonstrated minimal impacts on the development of antibiotic resistance in long-term. Notably, resistance to ampicillin was lower by 72% in comparison with a control after 90 days in the presence of biologically produced AgNPs, while resistance to tetracycline and kanamycin dropped to nearly negligible levels. In contrast, chemically synthesized AgNPs and ionic silver substantially increased antibiotic resistance in long-term, particularly to ampicillin and chloramphenicol, where resistance levels were 11 to 13 times higher than the controls, respectively. Chemically synthesized AgNPs caused a gradual rise in resistance, while ionic silver induced consistently elevated resistance throughout the study. Conclusions: These differences highlight the complex interplay between nanoparticle composition and bacterial adaptation. The findings suggest that biologically synthesized AgNPs are a promising environmentally friendly alternative, reducing bacterial resistance and mitigating the risks associated with silver-induced antibiotic resistance in soil ecosystems. They have greater potential for sustainable applications while addressing critical concerns about antimicrobial resistance and environmental safety.

## 1. Introduction

Silver nanoparticles (AgNPs) are among the most widely used nanomaterials today, with annual global production estimated at between 135 and 420 tons [1]. Their exceptional antimicrobial properties have led to applications across various industries, including medicine, pharmaceuticals, electronics, cosmetics, agriculture, and textiles. Chemical synthesis often involves reducing agents like sodium borohydride or sodium citrate to convert silver ions into nanoparticles. Biological synthesis utilizes enzymes from bacteria, fungi, or plants to reduce silver ions, often using plant extracts or bacterial cultures. The main difference is that chemical methods provide precise control over nanoparticle size and shape, while biological methods are more environmentally friendly but offer less control and scalability [2,3]. Despite their utility, the rapid increase in AgNP production and usage has raised concerns about environmental contamination, particularly in soil ecosystems.

AgNPs are known to exert toxic effects on soil bacteria even at low concentrations, which play critical roles in maintaining ecosystem functions, including nutrient cycling (carbon, nitrogen, and phosphorus), organic matter decomposition, as well as serving as a food source for other organisms [4]. The accumulation of AgNPs in soil represents a potential risk to bacterial communities, potentially disrupting ecological processes and soil health [5,6]. Long-term exposure to AgNPs can significantly alter the abundance of major bacterial phyla in soil, including Proteobacteria, Actinobacteria, and Firmicutes [4,7]. Given these risks, understanding the impact of AgNPs on soil bacteria is crucial, including their effects on promoting increased bacterial resistance to antibiotics.

AgNPs exhibit unique physical, chemical, and biological properties influenced by their size, shape, and surface characteristics, which can be tailored through different synthesis methods. Chemical and physical synthesis techniques are widely used, but they often require toxic chemicals and complex equipment [8,9]. In contrast, biological or “green” synthesis methods utilize plant extracts or microorganisms to produce AgNPs, offering a more environmentally friendly alternative [6,10]. These biologically synthesized nanoparticles have shown strong antimicrobial properties and may differ in environmental behaviour and stability compared to their chemically synthesized counterparts, as have been shown for microbial [11,12] and plant ecosystems [12]. However, studies that compare biological (other than antimicrobial) effects of chemically and biologically produced nanoparticles are very rare.

One critical area of investigation is the potential of AgNPs to induce bacterial resistance to antibiotics, a growing global concern. Previous studies have shown that exposure to various forms of silver, including nanoparticles or ionic silver, can promote the spread of antibiotic resistance genes and increase bacterial tolerance to multiple antibiotics [13,14]. However, the specific impact of biologically synthesized AgNPs on resistance remains underexplored.

This study aims to compare the effects of the production route on biological effects of AgNPs in the soil environment. It seeks to compare their antimicrobial properties and influence on the antibiotic resistance proliferation. By examining these factors, this research provides insights into the environmental behaviour of AgNPs and their potential risks, enabling the better assessment and management of their impact on soil ecosystems.

## 2. Results and Discussion

### 2.1. Characteristics of Used Silver Nanoparticles

Both biologically and chemically produced silver nanoparticles used in the experiments were characterized by TEM and UV-vis spectrometry (Table 1).

### 2.2. Effect of Silver Forms on Total Soil Bacteria

**Figure 1:**
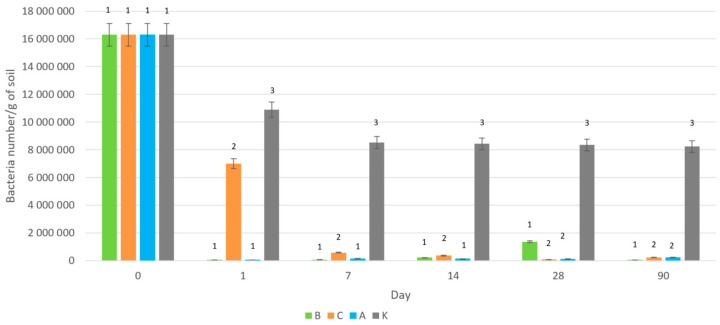
Changes in bacterial counts after the application of the studied forms of silver into soil systems: B—soil with added biologically produced nanoparticles, C—soil with added chemically produced nanoparticles, A—with ionic silver, K—control without silver (Groups 1, 2, and 3 are significantly different according to one-way ANOVA at *p* = 0.0026).

Silver application significantly affected soil bacterial populations across all silver forms studied. Biologically synthesized silver nanoparticles (B-AgNPs) and ionic silver exhibited the most profound effects, causing a 99.7% reduction in bacterial counts within the first day after the application. This dramatic decline was maintained throughout the experimental period, with negligible further reductions noted (Figure 1).

In contrast, chemically synthesized silver nanoparticles (C-AgNPs) caused a more gradual reduction, with a 57% decrease observed after one day and a 96% reduction within the first week. By the end of the 90-day experiment, only 1.5% of the initial bacterial population survived in the soil treated with chemically produced nanoparticles.

These findings confirm the strong antimicrobial effects of silver across all forms, with biologically synthesized AgNPs demonstrating the most immediate and pronounced toxicity. This aligns with previous studies, such as those by Chahara et al. [15], who compared the effects of various biologically and chemically synthesized AgNPs on bacterial strains. They observed that biologically synthesized AgNPs, especially those derived from plant extracts, exhibited superior antimicrobial activity compared to their chemically synthesized counterparts. This observation was further supported by studies from Mousavi-Khattat et al. [16] and Antony et al. [17], both of which highlighted the enhanced antibacterial efficacy of biologically synthesized AgNPs against multiple bacterial species. These results suggest that the synthesis method significantly influences the antimicrobial properties of AgNPs, likely due to differences in surface chemistry and particle size.

### 2.3. Impact of Silver Forms on Antibiotic Resistance

**Figure 2:**
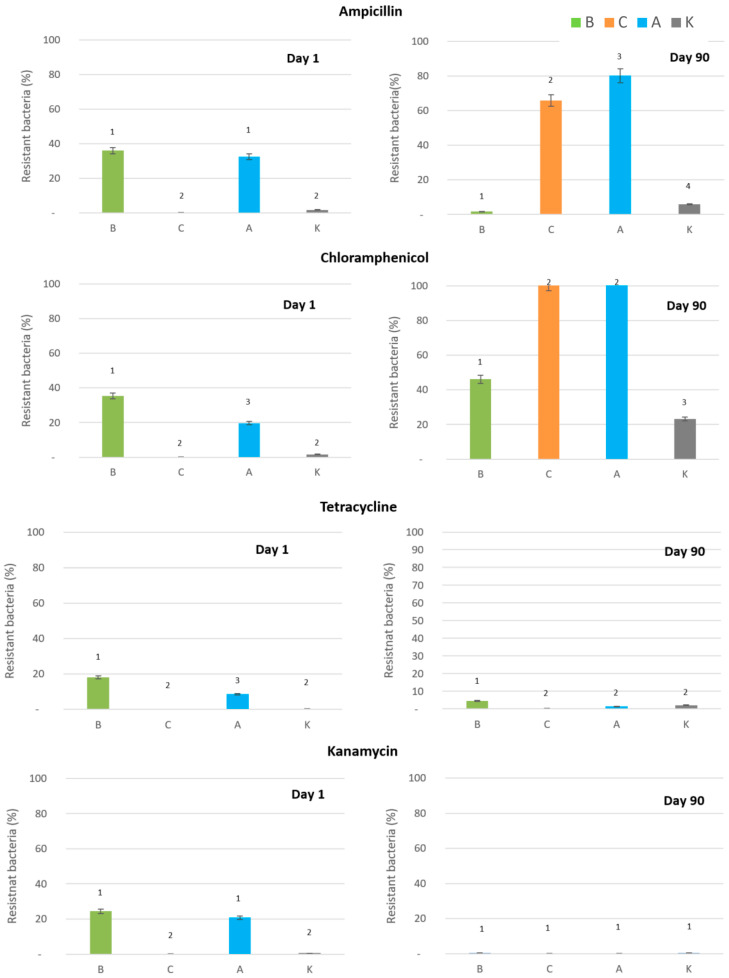
Presence of bacteria resistant to studied antibiotics as a percentage of the total bacterial count on the 1st and 90th day after the addition of different forms of silver: B—biologically and C—chemically produced AgNPs, A—Ag+ ions, K—control (no silver) (Groups 1, 2, 3, and 4 are significantly different according to one-way ANOVA at *p* = 0.0114).

Based on our results, the effect of AgNPs on antibiotic resistance proliferation significantly depends on the silver form, on the route, how the nanoparticles are prepared, as well as how long nanoparticles are present in the environment. After the first day (left part of Figure 2), in all cases, an increase in bacterial resistant to antibiotics was observed in soil systems treated with the biologically prepared nanoparticles and ionic silver following the most detrimental effects of the studied silver forms on the total bacterial counts. Chemically produced nanoparticles seemingly did not trigger antibiotic resistance in the soil ecosystem within the first day. However, the picture totally changed after 90 days (right part of Figure 2). While the percentage of bacterial resistance to studied antibiotics increased in the soil treated with silver ions significantly from 33 to 80% and from 20 to 100% for ampicillin and chloramphenicol, respectively, in the soil system treated with B-AgNPs, the picture was different. The percentage of bacteria resistant to ampicillin decreased from 36 to 2, and in the case of chloramphenicol, the percentage increased from 35 to 46%; however, this stayed the smallest in comparison with C-AgNPs and ionic silver where all isolated bacteria were resistant to these antibiotics. This shows that the the exposure time to silver ions/nanoparticles plays an important role in the antibiotic resistance proliferation and that the long-term effect may differ from the effect that is visible within the standard 24–48 h tests. The trend of antibiotic resistance reduction over time when B-AgNPs were tested is visible for all studied antibiotics; however, to explain it needs further study.

After the 90 days, biologically synthesized silver nanoparticles consistently showed the least impact, triggering 72% less ampicillin-resistant bacteria over 90 days compared to controls. Conversely, ionic silver and chemically synthesized AgNPs caused a substantial increase in antibiotic resistance, with resistance levels 11 to 13 times higher than in controls, respectively, by the end of the experiment. These findings indicate that silver’s effects on resistance development depend not only on its presence but also on its form.

For chloramphenicol, biologically synthesized AgNPs resulted in a modest twofold increase in resistance, while C-AgNPS and ionic silver caused a nearly fivefold increase. It only partially confirms previously published results that shows that ionic silver significantly elevates antibiotic resistance gene levels compared to AgNPs [14].

Tetracycline and kanamycin resistance in long-term observations showed negligible effects for all the studied silver forms. Biologically synthesized AgNPs and ionic silver both showed a decrease in the percentage of resistant bacteria over time. For example, the percentage of tetracycline-resistant bacteria dropped from 18% to 5% and from 9% to 1% with biologically synthesized AgNPs and ionic silver, respectively, after 90 days. A similar pattern was observed for kanamycin, where resistance levels fell from 24% to 0% with biologically synthesized AgNPs and from 21% to 0% with ionic silver. Chemically synthesized AgNPs and control samples did not trigger resistance to tetracycline and kanamycin throughout the study.

These findings corroborate previous studies highlighting the role of silver in promoting resistance, particularly in its ionic form. Kaweeteerawat et al. [13] demonstrated that sublethal doses of AgNPs increase resistance to multiple antibiotics, while Chen et al. [14] reported significant increases in resistance genes with ionic silver compared to AgNPs. Lu et al. [18] further emphasized that both silver forms accelerate horizontal gene transfer, with ionic silver exhibiting stronger effects. On the other hand, Guo et al. [19] found that the presence of silver nanoparticles in aquatic estuarine significantly attenuated the presence of antibiotic resistance genes in biofilms.

Overall, biologically synthesized AgNPs emerged as the most promising option for mitigating antibiotic resistance while maintaining antimicrobial activity, especially in the long term. Their minimal impact on the percentage of resistant bacteria, compared to the dramatic effects of ionic silver and chemically synthesized AgNPs, underscores their potential for safer applications in medicine and environmental management. Further studies are warranted to explain this behaviour and optimize nanoparticle formulations for practical use.

### 2.4. Bacterial Resistance to Silver Ions

**Figure 3:**
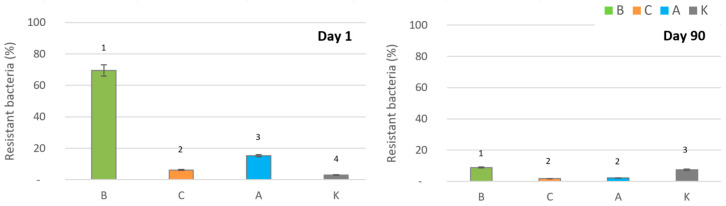
Presence of bacteria resistant to silver as a percentage of the total bacterial count on the 1st and 90th day after the addition of different forms of silver: B—biologically and C—chemically produced AgNPs, A—Ag+ ions, K—control (no silver) (Groups 1, 2, 3, and 4 are significantly different according to one-way ANOVA at *p* = 0.0092).

Resistance to silver ions after 90 days was generally low across all treatments, even in soil systems treated with ionic silver. This indicates that silver ion application did not select resistant bacterial strains during the experimental period. Interestingly, in soil systems treated with biologically synthesized AgNPs, the proportion of silver ion-resistant bacteria initially spiked to 70%, but gradually declined to 10% by the end of the experiment (Figure 3). Probably only bacteria resistant to silver were able to survive the fast toxic effect of B-AgNPs and ionic silver observed within the first day. In the case of C-AgNPs, the toxic effect was weaker as 7 days were necessary to reduce the total count of bacteria by 96%, so very little bacteria were able to survive and resistance to silver did not show.

In soil systems treated with ionic silver or chemically synthesized AgNPs, resistance levels remained consistently low, highlighting that biologically synthesized AgNPs uniquely influence the transient dynamics of silver ion resistance. These findings support the hypothesis that biologically synthesized AgNPs, while highly effective antimicrobial agents do not promote the long-term selection of resistant bacterial populations.

Only a small number of articles observed similar findings, suggesting that biologically synthesized silver nanoparticles (AgNPs) hold promise as potent antimicrobial agents with a low potential to induce silver resistance. Studies have shown that biologically produced AgNPs are effective against multidrug-resistant bacteria, such as *Pseudomonas aeruginosa* [20,21], and can inhibit the growth of various resistant pathogens [22,23]. Authors highlighted the unique properties of biologically synthesized silver nanoparticles, such as their smaller size and larger surface area compared to chemically synthesized ones. The higher long-term stability of B-AgNPs in comparison with C-AgNPs can also be a reason why biologically produced silver nanoparticles have different biological effects in soil ecosystems [24]. These properties may influence their interaction with bacteria and their potential to induce resistance. While further research is needed to fully understand the mechanisms behind this behaviour, these findings indicate that biologically synthesized AgNPs may offer a sustainable and environmentally friendly approach to combat antimicrobial resistance. This research highlights the importance of evaluating the environmental implications of AgNP use and identifying nanoparticle forms that minimize ecological disruption. Biologically synthesized nanoparticles emerge as a promising option, offering a balance between effective antimicrobial properties and ecological safety.

## 3. Materials and Methods

### 3.1. Soil Sampling

Soil samples were collected from meadow soil in Myslava, Košice, Slovakia (GPS: S48.721836, V21.203141) at 10 °C. Samples (1800 g) were taken from the topsoil (0–30 cm) at three triangulated points, sieved (2 Mm mesh), and stored at 4 °C. The sampling was realized fully in accordance with standard [ISO] STN ISO 10381-6 [25]. Classified as loamy, the soil had a moisture content of 34.1% WHCmax.

### 3.2. Preparation of Chemically and Biologically Synthesized Silver Nanoparticles

Silver nanoparticles were prepared chemically (C-AgNPs) by boiling a 10⁻^3^ M AgNO₃ solution, adding 1% sodium citrate, and refluxing for one hour. The colloidal solution appeared brown to grey, confirming AgNP formation [26]. Biologically synthesized AgNPs (B-AgNPS) were produced using extracts from microscopic green alga *Parachlorella kessleri* by the method described in Kadukova [15]. Both types of nanoparticles were freshly prepared before the experiments and used within two weeks after the preparation. Nanoparticles were stored in a refrigerator at 4 °C before use.

### 3.3. Soil Ecosystem Effect Study

Four beakers with 400 g of soil each were moistened to 50% WHC and incubated at 18 °C for seven days. After that time, biologically or chemically synthesized AgNPs, ionic silver, or distilled water (control) at 1 mg/kg were added into beakers following the experimental design in one dose and incubated in the dark. Nanoparticles were added into distilled water used to moisten the soil at the beginning of the experiment. Soil samples (5 g) were collected at intervals (1, 7, 14, 28, and 90 days) to study the effect on all present bacteria.

### 3.4. Isolation, Enumeration and Resistance Assay of Soil Bacteria

The soil sample (5 g) was mixed with 50 mL of phosphate-buffered saline, shaken for 30 minutes, and supernatant serially diluted. Dilutions of 10-2–10-5 were used for controls, and for samples with added silver forms, 100–10-2 was used. For each dilution, triplicates were used. Dilutions were plated on Tryptic Soy Agar (TSA) and incubated at ambient temperature for 48 hours to count colony-forming units (CFUs). Antibiotic susceptibility testing was performed using the agar dilution method [27]. Bacteria were tested for resistance to ampicillin (50 µg/mL), chloramphenicol (10 µg/mL), tetracycline (10 µg/mL), and kanamycin (30 µg/mL) on Mueller–Hinton agar. Plates were inoculated with bacterial suspensions and incubated for 48 hours. Resistance to silver ions (1 mg/L) was assessed on TSA plates with added Ag⁺ ions solution.

### 3.5. Statistical Analysis

Statistical analyses were performed using Social Statistics online software tool (https://www.socscistatistics.com/ (28 November 2024)).

## 4. Conclusions

The increased production and widespread use of silver nanoparticles (AgNPs) have raised concerns about their contribution to environmental contamination, par-ticularly in soil ecosystems. With their potent antibacterial properties, it is essential to understand the potential risks AgNPs pose to these ecosystems. This study evaluated the impact of silver nanoparticles produced by two routes, biological and chemical, and ionic silver on soil bacterial communities.

Our findings reveal that silver, in any form, significantly reduces bacterial popu-lations in soil. Biologically synthesized nanoparticles and ionic silver had the most pronounced toxic effects, causing substantial reductions in bacterial counts within just one day. In contrast, chemically synthesized nanoparticles showed a milder impact, likely due to differences in their toxicological properties. These results highlight the adverse effects of silver on soil ecosystems and demonstrate that the toxicity of nano-particles is influenced by their synthesis method. Additionally, silver application in-creased resistance to certain antibiotics, such as ampicillin and chloramphenicol, alt-hough resistance to tetracycline, kanamycin, and silver ions remained relatively un-changed.

Importantly, this study emphasizes the unique properties of biologically synthe-sized AgNPs. While they exhibit strong antimicrobial activity, they do not contribute to the development of silver resistance among soil bacteria—a critical finding for ad-dressing concerns about antimicrobial resistance. This underscores the need for further research to optimize their applications and assess their long-term ecological impact.

Overall, this research highlights the importance of evaluating the environmental implications of AgNP use and identifying nanoparticle forms that minimize ecological disruption. Biologically synthesized nanoparticles emerge as a promising option, of-fering a balance between effective antimicrobial properties and ecological safety.

## Figures and Tables

**Table 1:** Basic characteristics of biologically and chemically produced AgNPs.

	B-AgNP	C-AgNP
Size	9 ± 2 nm	20–90 nm
Shape	Spherical	Spherical
Surface	Protein biocorona	Citrate anions

For a detailed characterization of biologically produced silver nanoparticles, other methods, such as FT-IR and EDS analyses, were used; the results were published in Kadukova [15].

## Data Availability

The raw data supporting the conclusions of this article will be made available by the authors on request.
